# Molecular characterization of a novel GSTO2 of *Fasciola hepatica* and its roles in modulating murine macrophages

**DOI:** 10.1051/parasite/2022016

**Published:** 2022-03-22

**Authors:** Xifeng Wang, Chunguang Zhao, Guowu Zhang, Kai Zhang, Zhiyuan Li, Yunxia Shang, Chengcheng Ning, Chunhui Ji, Xianzhu Xia, Xuepeng Cai, Jun Qiao, Qingling Meng

**Affiliations:** 1 College of Animal Science & Technology, Shihezi University Shihezi Xinjiang 832003 China; 2 State Key Laboratory of Veterinary Etiological Biology, Lanzhou Veterinary Research Institute, Chinese Academy of Agricultural Sciences Lanzhou Gansu 730046 China

**Keywords:** *Fasciola hepatica*, GSTO2, Molecular characterization, Biochemical properties, Murine macrophages, Immunomodulatory role

## Abstract

Fascioliasis is an important zoonotic helminthic disease caused by *Fasciola hepatica* and poses a serious threat to global public health. To evade the immune response of its host (humans or animals), *F. hepatica* secretes various antioxidant enzymes such as glutathione transferase (GST) to facilitate its invasion, migration and parasitism *in vivo*. To investigate the biological functions of a novel omega-class GST (GSTO), the molecular features of GSTO2 of *F. hepatica* were analyzed by online software, and the biochemical properties *in vitro* of recombinant GSTO2 (rGSTO2) were dissected. Then, the regulatory roles of rGSTO2 protein in murine macrophages *in vitro* were further explored. The results revealed that the *GSTO2* gene encodes 254 amino acids, which harbor the characteristic N-terminal domain (βαβαββα) and C-terminal domain (α-helical) of the cytoplasmic GST superfamily. GSTO2 was mainly expressed in *F. hepatica* vitelline follicles, intestinal tract, excretory pores and vitelline cells, with thioltransferase and dehydroascorbate reductase activities. Moreover, rGSTO2 protein could be taken up by murine macrophages and significantly inhibit the viability of macrophages. In addition, rGSTO2 protein could significantly promote apoptosis and modulate the expression of cytokines in macrophages. These findings suggested that *F. hepatica* GSTO2 plays an important role in modulating the physiological functions of macrophages, whereby this protein might be involved in immunomodulatory and anti-inflammatory roles during infection. This study provided new insights into the immune-evasion mechanism of *F. hepatica* and may contribute to the development of a potential anti-inflammatory agent.

## Abstract (fr)

La fasciolase est une importante maladie helminthique zoonotique causée par *Fasciola hepatica*, qui constitue une menace sérieuse pour la santé publique mondiale. Pour échapper à la réponse immunitaire de son hôte (humain ou animal), *F. hepatica* sécrète diverses enzymes antioxydantes telles que la glutathion transférase (GST) pour faciliter son invasion, sa migration et son parasitisme *in vivo*. Pour étudier les fonctions biologiques d’une nouvelle GST de classe oméga (GSTO), les caractéristiques moléculaires de la GSTO2 de *F. hepatica* ont été analysées par un logiciel en ligne et les propriétés biochimiques *in vitro* de sa protéine recombinante (rGSTO2) ont été disséquées. Ensuite, les rôles régulateurs de la protéine rGSTO2 sur les macrophages murins *in vitro* ont été explorés plus avant. Les résultats ont révélé que le gène GSTO2 code pour 254 acides aminés, qui abritent le domaine N-terminal caractéristique (βαβαββα) et le domaine C-terminal (α-hélicoïdal) de la superfamille GST cytoplasmique. Chez *F. hepatica*, GSTO2 était principalement exprimée dans les follicules vitellins, le tractus intestinal, les pores excréteurs et les cellules vitellines, avec des activités de thioltransférase et de déhydroascorbate réductase. De plus, la protéine rGSTO2 a pu être absorbée par les macrophages murins et inhiber de manière significative la viabilité des macrophages. Enfin, la protéine rGSTO2 a pu favoriser de manière significative l’apoptose et moduler l’expression des cytokines dans les macrophages. Ces résultats suggèrent que la GSTO2 de *F. hepatica* joue un rôle important dans la modulation des fonctions physiologiques des macrophages, cette protéine pouvant être impliquée dans des rôles immunomodulateurs et anti-inflammatoires au cours de l’infection. Cette étude a fourni de nouvelles informations sur le mécanisme d’évasion immunitaire de *F. hepatica* et pourrait contribuer au développement d’un agent anti-inflammatoire potentiel.

## Introduction

*Fasciola hepatica* is a food-borne parasite that parasitizes ruminants, humans and wild animals, and that is widespread around the world. This worm can lead to acute and chronic fascioliasis with a series of complications such as nutritional disorders and systemic toxicity after infection [32]. In ruminants, *F. hepatica* often causes chronic infection, and is responsible for major economic losses in animal production. What’s more, *F. hepatica* can infect humans through metacercariae-contaminated water and edible aquatic vegetation, resulting in human fasciolosis, which is now considered an emerging or re-emerging neglected disease. So far, it is estimated that about 2.6 to 17 million humans are assumed to be infected [7, 17], which has posed serious threats to public health.

During helminth infections, *F. hepatica* can release various antigenic molecules (e.g., tegumental antigen, Teg; excretory secretory products, ESPs), whereby these molecules can interact with host immune cells (macrophages, dendritic cells, T lymphocytes, etc.) to modulate the host immune responses [1, 13, 16], thus exerting their immunomodulatory functions. As one of the important components of *F. hepatica* ESP antigens, glutathione transferase (GST) is a multifunctional enzyme, accounting for 4% of the total soluble protein and with a widespread tissue distribution [8, 26]. GST can not only detoxify both exogenous and endogenous toxins in the host bile environment [8], but also prevent the secondary products of lipid peroxidation from immune-induced free radical attacks [5], which suggests that this enzyme is not only obviously responsible for general cellular functions, but also functions as an immune response protein in helminth infection [8]. To date, at least seven classes of mammalian GSTs (Alpha, Theta, Zeta, Mu, PI, Sigma and Omega) have been identified in the GST superfamily [9, 34], whereas four classes of GST (Omega, Mu, Sigma and Zeta) have been found in *F. hepatica* based on biochemical and amino acid sequence differences [29]. Among them, omega-class GSTs (including GSTO1 and GSTO2) were identified by expressed sequence tagging (EST) and sequence comparison [4], which harbored an extended sequence of 19 amino acids at the N-terminal end with a proline-rich domain [9, 38]. Previous studies had reported that human GSTO may be involved in the cellular detoxification and excretion of many physiological and xenobiotic substances [40], implying that GSTO may possess similarly important functions in *F. hepatica*.

Although an omega-class GST (GSTO2) has already been identified in *F. hepatica*, the physiological function of GSTO2 is still unknown. The main objective of this study was to understand the molecular characteristics and biochemical properties of the newly discovered GSTO2 in *F. hepatica*, investigate its immunomodulatory roles in murine macrophages, and explore the immune-evasion mechanisms of *F. hepatica*.

## Materials and methods

### Ethics statement

BALB/c mice used in this study were fed adequate food and water in a clean and comfortable environment. The experiments were carried out in accordance with the guidelines issued by the Ethics Committee of Shihezi University (No. A20180126).

### Parasites, sera and cells

The adult flukes were collected from the livers of sheep in a Xinjiang Urumqi slaughterhouse. After washing them with phosphate buffered saline (PBS, 0.01 M, pH 7.2–7.4) three times, and then following Haridwal [24] and Giovanoli Evack’s methods [18], the worms were identified as *F. hepatica* by morphological and molecular biological techniques. Anti-*F. hepatica* sera were collected from sheep whose livers were found to be infected with *F. hepatica* at the abattoir and negative sera were collected from captive 6-month-old sheep. RAW264.7 murine macrophages were cultured in DMEM culture medium supplemented with 10% fetal bovine serum (FBS) at 37 °C in 5% CO_2_.

### Cloning and molecular characterization of the *GSTO2* gene

Based on the *GSTO2* gene sequence of *F. hepatica* deposited in GenBank (accession number: MH230107), a pair of oligonucleotide primers was designed as follows: FP (5′–GGAATTCATGATGGCCGTTGTGGGC–3′, where the *Eco*R I site was introduced into the 5′ end), RP (5′–CCCTCGAGTCAAACGATATATCCGGATTTGG–3′, where the *Xho* I site was introduced into the 5′ end). Total RNA was extracted from *F. hepatica* using an RNAqueous^®^-Micro Kit (Thermo Fisher, Waltham, MA, USA), and then reversely transcribed into cDNA by a PrimeScript^TM^ RT reagent Kit (TaKaRa Biotech, Dalian, China). The *GSTO2* gene was amplified by PCR using *F. hepatica* cDNA as a template. The PCR reaction was subjected to 1 cycle at 95 °C for 5 min and 35 cycles (at 94 °C for 40 s, 64 °C for 40 s, 72 °C for 1 min), followed by extension at 72 °C for l0 min. The amplified products were visualized by electrophoresis with 1% agarose gel (Biowest agarose, Spain) and purified according to the instructions of a Gel Extraction kit (Omega Biotek, Norcross, GA, USA). The target fragment was ligated into the pMD-19T vector (Takara Biotech, Dalian, China) and then transformed into *Trans5α* chemically competent cells (TransGen Biotech, Beijing, China), the positive transformants were screened by PCR and sequenced by Sangon Biotech (Shanghai, China). Subsequently, the plasmid was extracted from the positive transformant with a FastPure Plasmid Mini kit (Vazyme Biotech, Nanjing, China) and digested with *Eco*R I and *Xho* I restriction endonuclease, and then cloned into the pET-28a (+) vector (Invitrogen, Carlsbad, CA, USA) to generate recombinant plasmid pET-GSTO2.

### Expression of GSTO2 protein and polyclonal anti-GSTO2 sera production

In brief, pET-GSTO2 plasmid was transformed into *E. coli* BL21 (DE3) competent cells (TransGen Biotech) for expression. After the transformants were cultured in LB medium, the bacteria were induced by 1 mMol/L isopropyl-β-D-thiogalactopyranoside (IPTG, Biotopped Biotech, Beijing, China) for the expression of recombinant GSTO2 (rGSTO2) protein. The rGSTO2 protein was purified using HisPur™ Ni-NTA Spin Columns (GE Healthcare, Chicago, IL, USA), and the purified protein was dialyzed with PBS (0.01 M, pH 7.4) at 4 °C, followed by SDS-PAGE and western blot analysis. Similarly, the empty pET-28a (+) plasmid (Invitrogen) was transformed into *E. coli* BL21(DE3), and then pET-28a protein (P28a) was prepared and purified using the same method as that of rGSTO2 protein. Then endotoxins were removed from the dialyzed proteins using an EtEraser^TM^ HP High Performance Endotoxin Removal Kit (Bioendo, Xiamen, China) and detected by a ToxinSensor^TM^ Chromogenic LAL Endotoxin Assay Kit (GenScript Biotech Corp, Nanjing, China) until endotoxin in rGSTO2 protein was lower than 0.1 EU/mL, which could be used for subsequent experiments.

To prepare polyclonal sera, 100 μg rGSTO2 or P28a protein were mixed with Freund’s complete adjuvant (1:1, Sigma-Aldrich, St. Louis, MO, USA), respectively and then injected into 6-week-old female BALB/c mice subcutaneously [27]. Two weeks after the first immunization, the mice were immunized with the same concentration of protein mixed with Freund’s incomplete adjuvant (1:1, Sigma-Aldrich), and the third booster immunization was performed 7 days after the immunization interval. Finally, the blood was collected from immunized mice 10 days after the last immunization, and the sera against rGSTO2 or P28a were separated by centrifugation at 2,500 rpm for 15 min, respectively. Meanwhile, the sera from naïve mice were used as negative control and stored at −20 °C until use.

### Immunofluorescence analysis of GSTO2 protein

To analyze the expression localization of GSTO2 in *F. hepatica,* immunofluorescence staining was performed on sections of adult *F. hepatica*. Briefly, an adult worm of *F. hepatica* was fixed in 4% paraformaldehyde at room temperature for 24 h and then the sections were prepared according to conventional techniques [22, 23]. The sections were incubated with mouse anti-rGSTO2 IgG or naïve mouse serum (control, 1:200) overnight at 4 °C, followed by incubation with FITC-conjugate goat anti-mouse IgG (1:1500, Abcam, UK) for 1 h at 37 °C. Then, the anti-fluorescence quenching blocking solution (Beyotime, Shanghai, China) was used to seal the sections for observation under a fluorescence microscope (Zeiss, Jena, Germany).

### Biochemical characterization of rGSTO2

rGSTO2 activities were determined by spectrophotometry using a panel of substrates [21]. The GST activity of 1-chloro-2, 4-dinitrobenzene (CDNB, Sigma-Aldrich) on rGSTO2 protein was measured at 340 nm absorbance. The reaction system was composed of 0.6 mM CDNB, 6 mM glutathione (GSH, Sigma-Aldrich) and 10 μg rGSTO2 protein, and the reaction was carried out in 100 mM phosphate buffer (pH 7.4). Furthermore, the activity of dehydroascorbate reductase (DHAR) was assayed in potassium phosphate buffer (50 mM, pH 7.4) containing 1 mM GSH, 0.25 mM dehydroascorbic acid (DHA, Sigma-Aldrich) and 10 μg rGSTO2 protein [39]. Thioltransferase activity was analyzed in potassium phosphate buffer (50 mM, pH 7.4) containing 0.2 mM NADPH, 0.5 mM GSH, 2 mM hydroxyethyl disulfide (HED) and 10 μg rGSTO2 protein [3]. The maximum reaction rate (*V*_max_) and Michaelis constant (*K*_m_) at the same temperature and pH were calculated according to the Michaelis–Menten equation [36].

### Optimum pH and temperature and kinetic analysis

The effects of pH and temperature on enzyme activity were analyzed by standard DHAR assay, and the optimum pH and temperature was determined. The DHAR kinetic parameters of the rGSTO2 were determined using a GSH range of 0–5 mM and a fixed DHA concentration of 0.25 mM. Similarly, the apparent *K*_m_ and *V*_max_ values for DHA were determined using a DHA range from 0 to 0.25 mM and a fixed GSH concentration of 1 mM. All assays were measured independently in triplicate at pH 7.5 and 25 °C.

### Analysis of interaction between rGSTO2 and RAW264.7 macrophages *in vitro*

Briefly, RAW264.7 macrophages were cultured in DMEM culture medium supplemented with 10% FBS in a humidified atmosphere of 5% CO_2_ at 37 °C. After the cells grew into a monolayer, the cells were transferred to a 6-well microplate (10^5^ cells/mL), and then rGSTO2 (20 μg/mL) or P28a protein and PBS (control group) were added to each well and co-incubated with the cells for 2 h of 5% CO_2_ at 37 °C. Cells were fixed with 4% paraformaldehyde at ambient temperature for 15 min, washed three times with PBS (5 min each time) and then blocked with 4% BSA in PBS at 37 °C for 1 h. Cells were incubated with mouse anti-rGSTO2 IgG, anti-P28a IgG and naïve serum (1:500 dilution) at 4 °C overnight, respectively followed by staining with Cy3-conjugated goat anti-mouse IgG (1:500, Abcam, UK) at 37 °C for 1 h. Then, the nuclei of cells were stained by 2-(4-Amidinophenyl)-6-indolecarbamidine dihydrochloride (DAPI, Beyotime, Shanghai, China) for 5 min. Finally, these cells were visualized using a confocal microscopy (Leica Microsystems GmbH, Wetzlar, Germany).

### Effects of rGSTO2 on cell viability of macrophages

The density of RAW264.7 macrophages was adjusted to 3 × 10^4^ cells/mL and the cells then plated on 96-well microplates, to which different concentrations of rGSTO2 (5 μg/mL, 10 μg/mL, 20 μg/mL, 40 μg/mL, and 80 μg/mL), PBS or 100 ng/mL lipopolysaccharide (LPS, Sigma-Aldrich) [28] were added, respectively. After incubation for 24 h, these cells were incubated with CCK-8 solution (Abcam, UK) at 37 °C for 4 h and then the OD_450nm_ was measured using a microplate reader (Thermo, Waltham, MA, USA). All tests were independently repeated three times.

### Effects of rGSTO2 on apoptosis of macrophages

Briefly, the density of RAW264.7 macrophages was adjusted to 10^5^ cells/mL and then plated on 6-well microplates for culture. Then, the different concentrations of rGSTO2 (5 μg/mL, 10 μg/mL, 20 μg/mL, 40 μg/mL, 80 μg/mL), PBS or 100 ng/mL LPS were added and cultured at 37 °C for 24 h, respectively. After the cells were washed twice with PBS and then digested with Accutase Enzyme (Lianke Biotech, Hangzhou, China) for 2 min, cells were collected and re-suspended with binding buffer, followed by apoptosis assay according to the instructions of the Annexin V-FITC/PI apoptosis kit (Lianke Biotech, Hangzhou, China). Annexin V-FITC and propidium iodide (PI) were added to each tube and vortexed gently, incubated in darkness at room temperature for 5 min and then detected by flow cytometry (BD Biosciences, Franklin Lakes, NJ, USA).

### Impacts of rGSTO2 on the expression of cytokine in macrophages

The density of RAW264.7 macrophages was adjusted to 10^6^ cells/mL and the cells then plated on 6-well microplates for culture, in which macrophages were treated with rGSTO2 (10 μg/mL) alone for 24 h or pretreated with rGSTO2 protein (10 μg/mL) alone for 24 h and then treated with LPS (100 ng/mL) for 12 h, respectively. Cells treated with PBS or LPS (100 ng/mL) were used as controls. Cells were washed with PBS and digested with trypsin-EDTA solution (Biosharp, Anhui, China), and then total RNA was extracted using an RNAqueous^®^-Micro Kit (Thermo Fisher). One μg of total RNA was reversely transcribed into cDNA according to the PrimeScript™ RT reagent Kit (TaKaRa Biotech, Dalian, China). Primers for real-time quantitative PCR were designed and synthesized (Table 1), and the target genes were normalized to the *GAPDH* housekeeping gene. After that, real-time quantitative PCR amplification was performed according to the Fast Start Universal SYBR Green Master (ROX) (Roche, Basel, Switzerland) instructions. The raw cycle thresholds (Ct) were obtained from LightCycler Application and then relative mRNA expression was calculated using the comparative Ct method with the formula 2^−▵▵CT^ [31].

**Table 1:** Primers used in real-time quantitative PCR.

Target gene	Primer name	Primer sequence (5′ → 3′)	Reference sequence (Acc. No./ID)
IL-6	F1	TCTGCAAGAGACTTCCATCC	M24221.1
	R1	CAATCAGAATTGCCATTGC	
IL-10	F2	ATGCCTGGCTCAGCACTGC	NM_010548.2
	R2	GGAGTCGGTTAGCAGTATGTTG	
IL-12	F3	GTAAATGTTAAATGCCCGCAG	M86671.1
	R3	GTACCTACGCAGCCCTGATTG	
IL-1β	F4	CAGCAGCACATCAACAAGAG	NM_008361.4
	R4	GTCTAATGGGAACGTCACACA	
INF-γ	F5	CGCTACACACTGCATCTTG	NM_008337.4
	R5	GCTGATGGCCTGATTGTC	
TNF-α	F6	GCACAGAAAGCATGATCCG	NM_001278601.1
	R6	TGAGTGTGAGGGTCTGGGC	
TGF-β	F7	CAGATCCTGTCCAAACTAAG	NM_011577
	R7	GCACTGCTTCCCGAATGTC	

### Statistical analysis of data

SPSS and GraphPad Prism software (SPSS Inc., Chicago, IL, USA) were used for statistical analysis of the data. All results were expressed as mean ± SD. One-way and two-way analysis of variance (ANOVA) were used to compare the statistical differences under different conditions, whereas a Tukey test was used for multiple comparison analysis. *P*-value < 0.05 was considered to be statistically significant.

## Results

### Amplification and molecular characterization of the *GSTO2* gene

**Figure 1:**
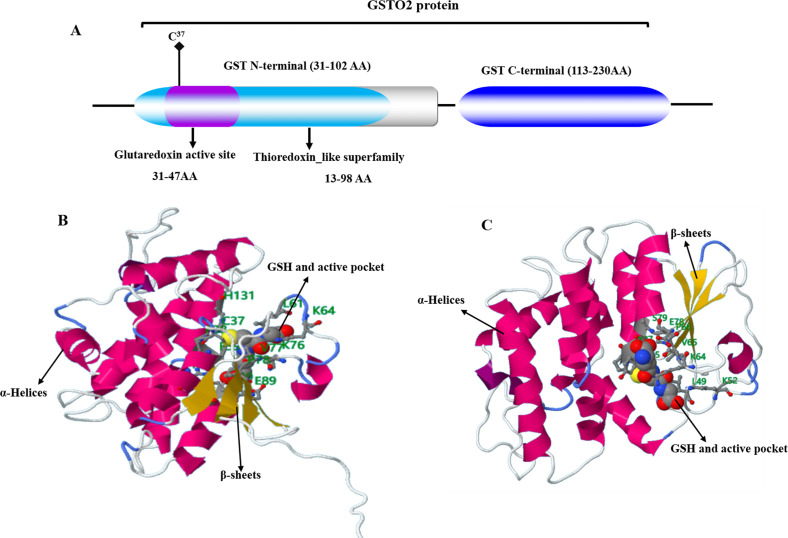
Molecular characteristics of omega-class GST of *F. hepatica.* (A) Schematic structural composition of the domains of GSTO2 protein. (B) and (C) represent the 3D structure models of GSTO2 and GSTO, respectively, which were built by I-TASSER. α-helices (red band) and β-sheets (yellow band) were connected by coils to form a stable tertiary structure. Key amino acids are marked by green letters.

**Supplementary Figure 2:**
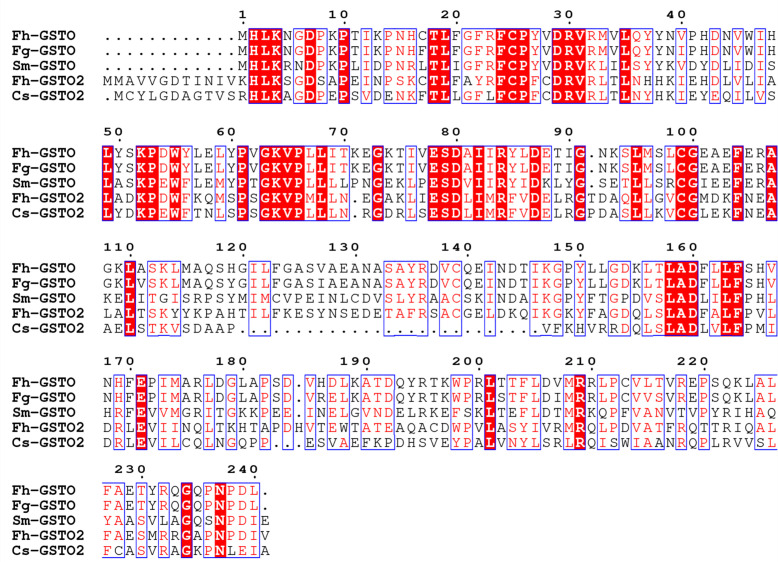
Comparisons of the amino acid sequence of GSTO2 protein of *F. hepatica* with other trematode GSTO. The red shadow indicates that the sequence consistency is 100%, while similar residues are shown as blue box. Fh: *Fasciola hepatica* (AFX98104.1); Fg: *Fasciola gigantica* (JX157881.1); Sm: *Schistosoma mansoni* (AAO49385.1); Cs: *Clonorchis sinensis* (KX163089.1).

The *GSTO2* gene of *F. hepatica* was 765 bp in length, encoding 254 amino acids, which contain one conserved cysteine residue (C^37^). The GSH binding site was composed of 12 amino acids (C^37^, P^38^, F^39^, R^42^, L^61^, K^64^, K^76^, V^77^, P^78^, E^89^, L^90^, and H^131^) and glutoxigenin active sites (31–47 AA) were found in the amino acid sequence of the GSTO2 protein (Supplementary Fig. S1). GSTO2 harbored two typical domains of the cytoplasmic GST superfamily, namely GST_N-terminal thioredoxin-like domain (31–102 AA, βαβαββα) and GST_C-terminal α-helix domain (113–230 AA) (Fig. 1A). However, there was no signal peptide in GSTO2. Compared the typical GSTO (Fig. 1C), GSTO2 was similarity to its tertiary structure, but there were some fine differences in spatial structures such as the GSH binding site and active site (Fig. 1B), which was expected to possess different substrate specificity [4]. However, *F. hepatica* GSTO2 shared 46.46% identities with GSTO2 (KX163089.1) of *Clonorchis sinensis* in the amino acid sequence, but it only shared 41.18% identities with GSTO of *F. hepatica* (Supplementary Fig. S2).

### Expression and localization analysis of the GSTO2 protein

**Figure 2:**
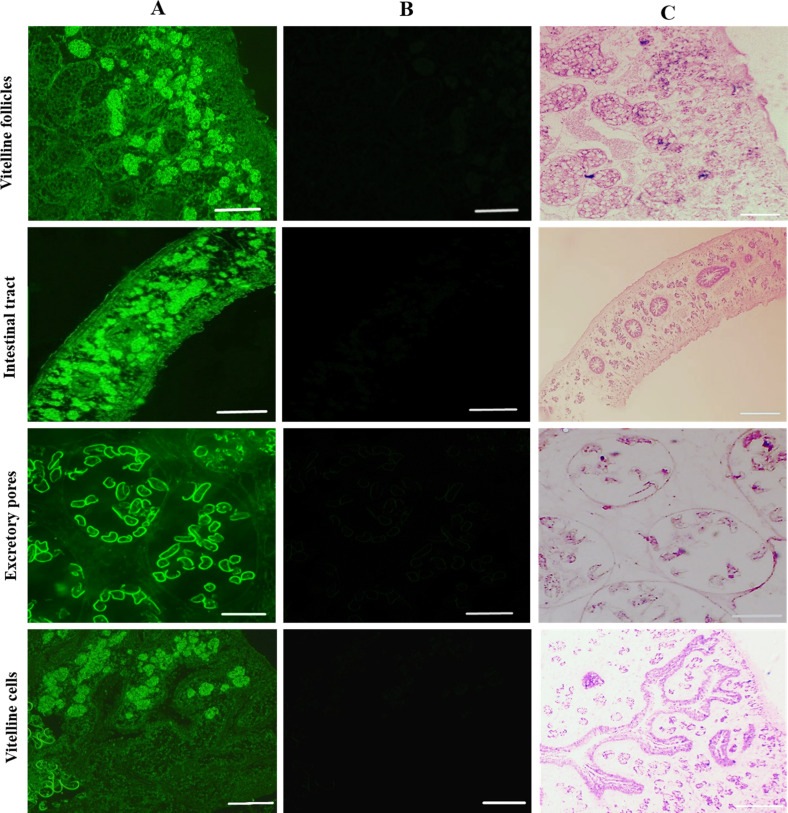
Immunofluorescence localization assay of GSTO2 protein expressed in *F. hepatica.* (A) Adult sections were incubated with mouse anti-rGSTO2 IgG (1:200) and subsequently incubated with FITC-conjugated goat anti-mouse IgG (1:1500 dilutions). (B) The same sections as column (A) were incubated with naïve mouse serum (1:200, negative control) and then incubated with FITC-conjugated goat anti-mouse IgG (1:1500 dilutions). (C) HE staining of sections of adult *F. hepatica*, scale-bars: 50 μm.

**Supplementary Figure 3:**
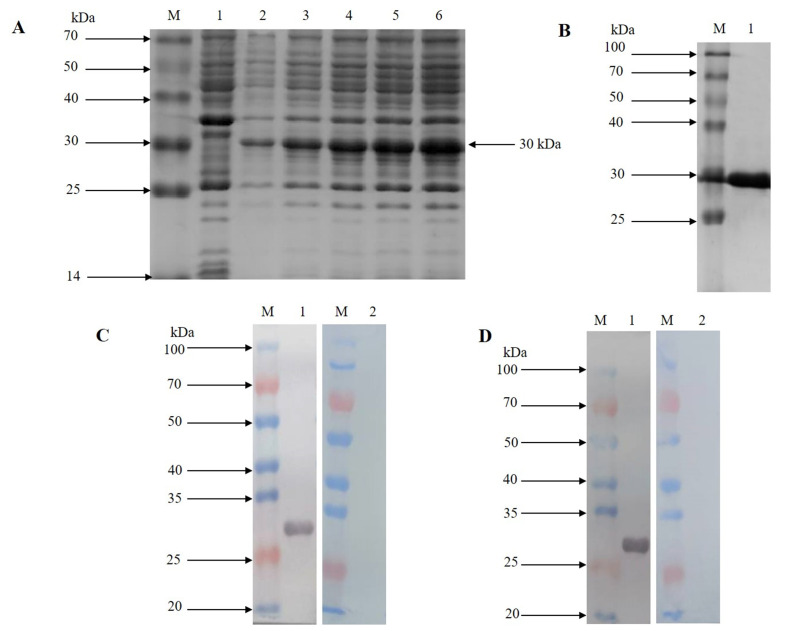
Identification of rGSTO2 protein of *F. hepatica* by SDS-PAGE and western blot. (A) Lane M, protein marker; lane 1, Sonicated supernatant of cultures after transformation of *E. coli* with the empty pET-28a plasmid; lane 2, lysate precipitation of rGSTO2 after induction for 6 h; lane 3-6, lysate supernatant of rGSTO2 at 2, 4, 6 and 8 hours induced by IPTG. (B) Lane M, protein marker; lane 1, purified rGSTO2 protein. (C) Lane M, protein marker; lane 1 and lane 2 were loaded with rGSTO2 protein and then incubated with sheep anti-*F. hepatica* sera or normal sheep serum, then incubated with HRP-conjugated rabbit anti-sheep IgG. (D) Lane M, protein marker; lane 1 and lane 2 were loaded with rGSTO2 protein and then incubated with mouse anti-rGSTO2 IgG or naïve mouse serum, respectively, and then incubated with HRP-conjugated goat anti-mouse IgG.

A recombinant GSTO2 protein (rGSTO2) with a molecular weight about 30 kDa was detected by SDS-PAGE, which reacted specifically with anti-*F. hepatica* sera or anti-rGSTO2 sera (Supplementary Fig. S3). As shown in Figure 2, rGSTO2 was mainly expressed in the vitelline follicles, intestinal tract, excretory pores and vitelline cells of adult *F. hepatica*, which was generally consistent with the expression localization of GSTO protein in *Caenorhabditis elegans* [6] and *Clonorchis sinensis* [25].

### Biochemical characterization of rGSTO2

**Figure 3:**
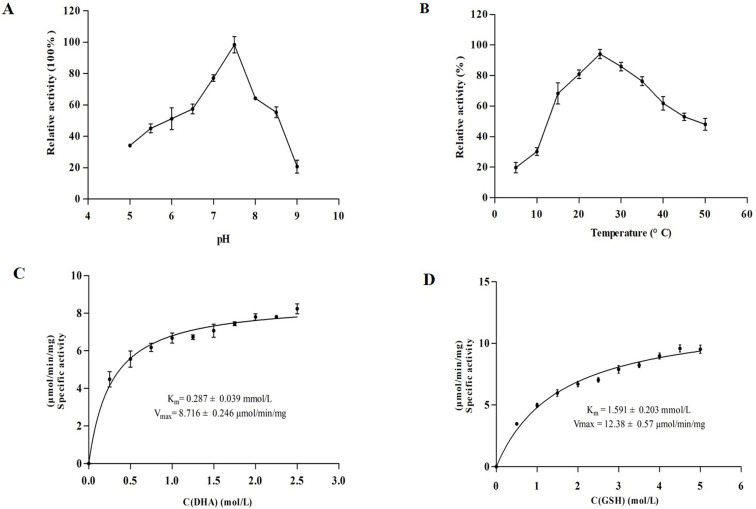
Determination of biochemical properties of GSTO2 of *F. hepatica*. (A) and (B) show the effects on catalytic activity of rGSTO2 protein measured at different pH (5.0–9.0) and different temperatures (5–50 °C) in potassium phosphate buffer (50 mM, pH 7.4) containing 1 mM GSH and 0.25 mM DHA, respectively. (C) and (D) represent the apparent *K*_m_ and *V*_max_ of rGSTO2 protein measured with GSH and DHA as substrates at different concentrations of DHA (0–2.5 mM) and GSH (0–5 mM) at 25 °C, pH 7.5, respectively.

The enzymatic activity of rGSTO2 against CDNB (0.22 ± 0.008 μMol/min/mg) was weak but with strong thioltransferase (TTas) activity (4.04 ± 0.15 μMol/min/mg). Since rGSTO2 exhibited very weak detectable activity against typical GST substrates, the assays associated with optimal enzymatic pH, optimum temperature, and kinetic parameters were all determined by DHAR assay. The results showed a maximum DHAR activity at pH 7.5 (Fig. 3A) and an optimum temperature of 25 °C (Fig. 3B). The enzymatic reaction catalyzed by rGSTO2 was fitted to a Michaelis–Menten kinetic model (Figs. 3C and 3D) by non-linear regression, the apparent *K*_m_ values for DHA and GSH were found to be 0.287 ± 0.039 mMol/L and 1.591 ± 0.203 mMol/L, respectively. The apparent *V*_max_ was 12.38 ± 0.57 μMol/min/mg, which was determined by a GSH range of 0–5 mM and fixed DHA concentration.

### Immunofluorescence assay

**Figure 4:**
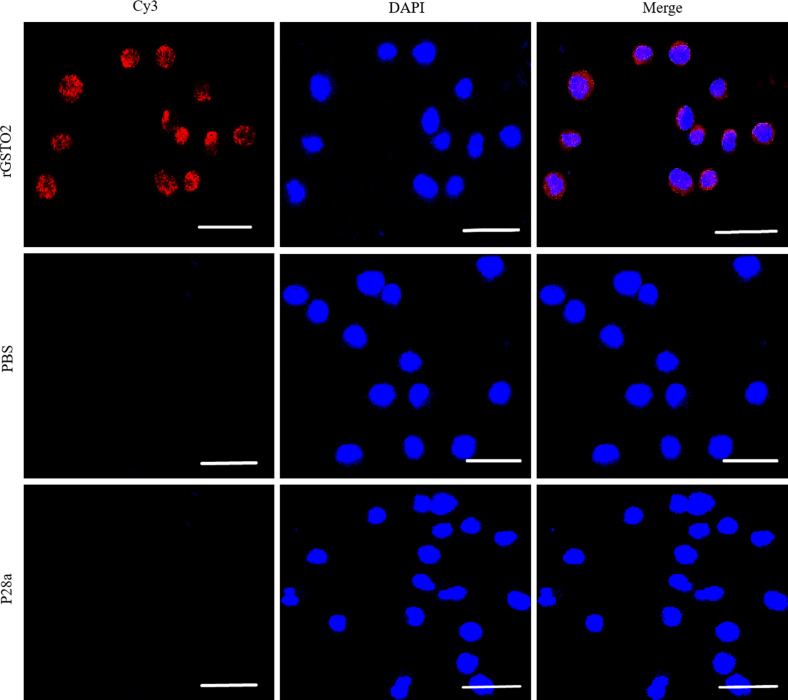
Localization of *F. hepatica*-derived rGSTO2 protein to the RAW264.7. macrophages surface. The macrophages were respectively pre-treated with rGSTO2, PBS or P28a protein, and the cells were then incubated with mouse anti-rGSTO2 IgG, naïve mouse serum or mouse anti-P28a IgG as primary antibody, followed by Cy3-conjugated goat anti-mouse IgG as the secondary antibody. The red fluorescence on the surface of cells indicated that the target protein (rGSTO2 protein) is stained (Cy3) and the cell nuclei was stained by DAPI (blue), while merge represents the overlap of the red and blue fluorescence channels. No fluorescence was observed in PBS or P28a control groups. Scale-bars: 10 μm.

By incubating rGSTO2-treated RAW264.7 macrophages with specific anti-rGSTO2 IgG, we detected uniform distribution and localization of the red Cy3 dye on the membrane of macrophages. On the contrary, no red fluorescence signal was observed in macrophages incubated with PBS or P28a protein (Fig. 4), which indicated that target protein rGSTO2 could be taken up by RAW264.7 macrophages *in vitro*.

### Effects of rGSTO2 on cell viability of macrophages

**Figure 5:**
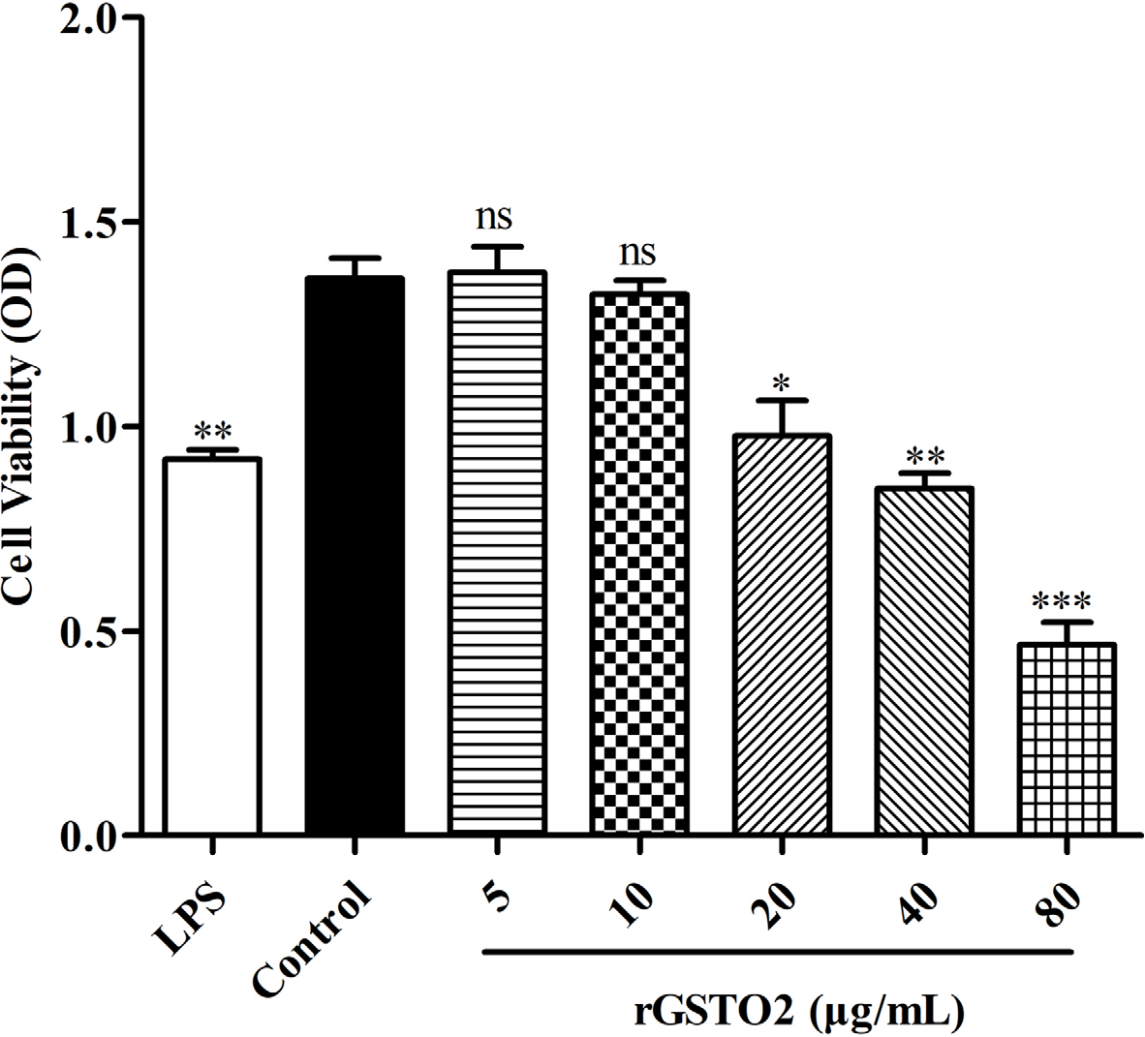
Viability assay of RAW264.7 macrophages stimulated by rGSTO2 protein *in vitro.* Macrophages treated with LPS or PBS were used as positive or negative controls, respectively and the cells were treated with rGSTO2 protein at different concentrations (5–80 μg/mL) for 24 h, and then the cell viability was measured by a CCK-8 assay. Ns means that the difference is not significant, ** *p* < 0.01 and ***p < 0.001 versus the negative control. All data are expressed as means ± SD (*n* = 3).

As shown in Figure 5, rGSTO2 did not modify macrophage viability at low concentrations (5 μg/mL and 10 μg/mL), but as the concentration increased, rGSTO2 protein significantly repressed the viability of RAW264.7 macrophages. Therefore, we chose 10 μg/mL rGSTO2 as an appropriate concentration for the following experiments.

### rGSTO2 induces apoptosis of RAW264.7 macrophages

**Figure 6:**
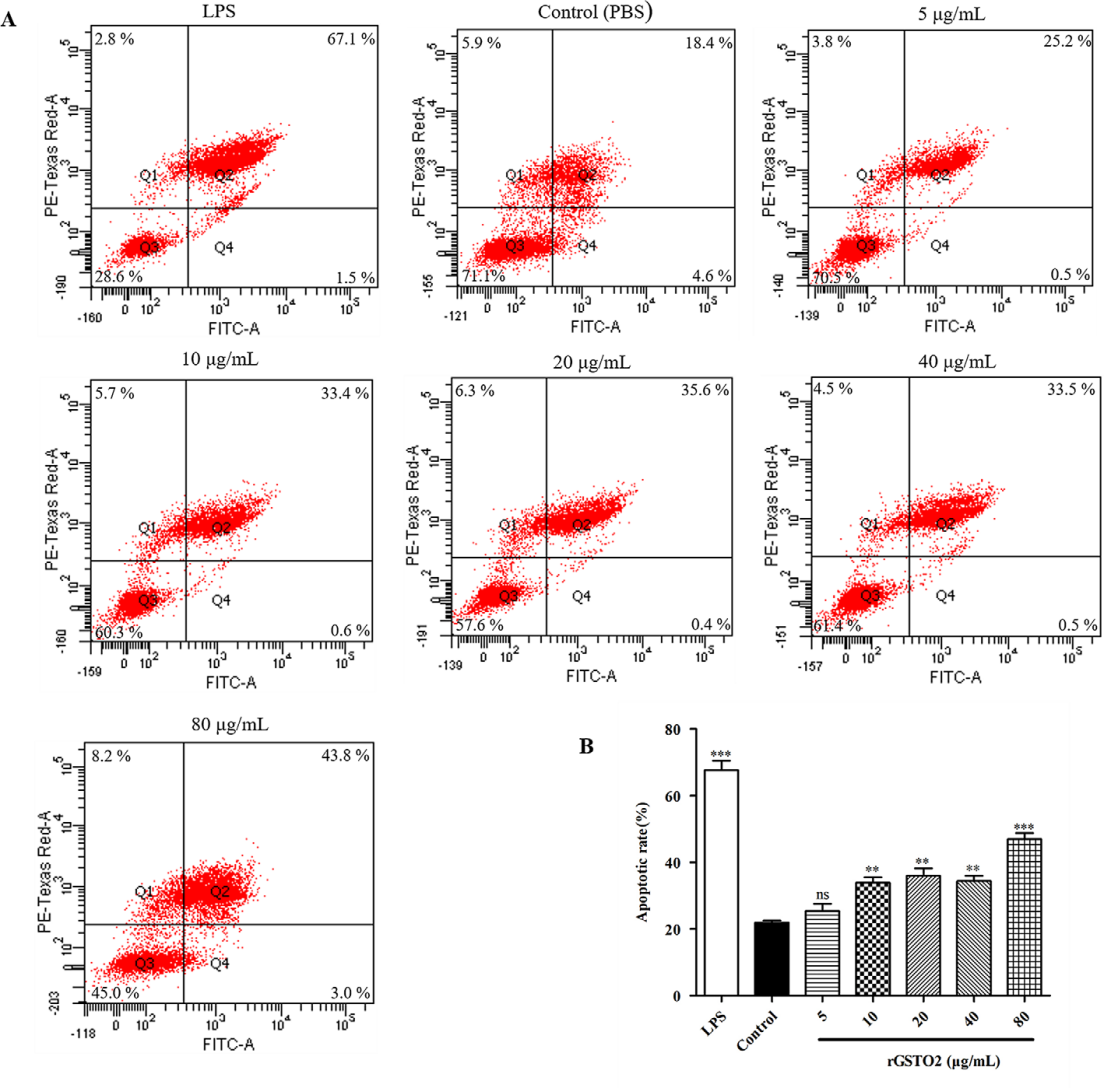
Effects of rGSTO2 on apoptosis of RAW264.7 macrophages. (A) RAW264.7 macrophages were stimulated with rGSTO2 and then stained with Annexin V and propidium iodide (PI), followed by the detection of apoptosis using flow cytometry. (B) Apoptosis rate of RAW264.7 macrophages stimulated by different concentrations of rGSTO2; Ns means that the difference is not significant, ** *p* < 0.01 and ***p < 0.001 indicates a significant difference compared to the control group. All data are expressed as means ± SD (*n* = 3).

After treatment with different concentrations of rGSTO2 for 24 h, the cells were stained with Annexin V and PI and then analyzed by flow cytometry. As shown in Figure 6, compared with the control group, the 5 μg/mL group had no significant effect on the apoptosis of RAW264.7 macrophages, but when the concentration of rGSTO2 was greater than 10 μg/mL, it significantly promoted macrophage apoptosis (**p < 0.01) in a dose-dependent manner.

### Impacts of rGSTO2 on the cytokine expression of RAW264.7 macrophages

**Figure 7:**
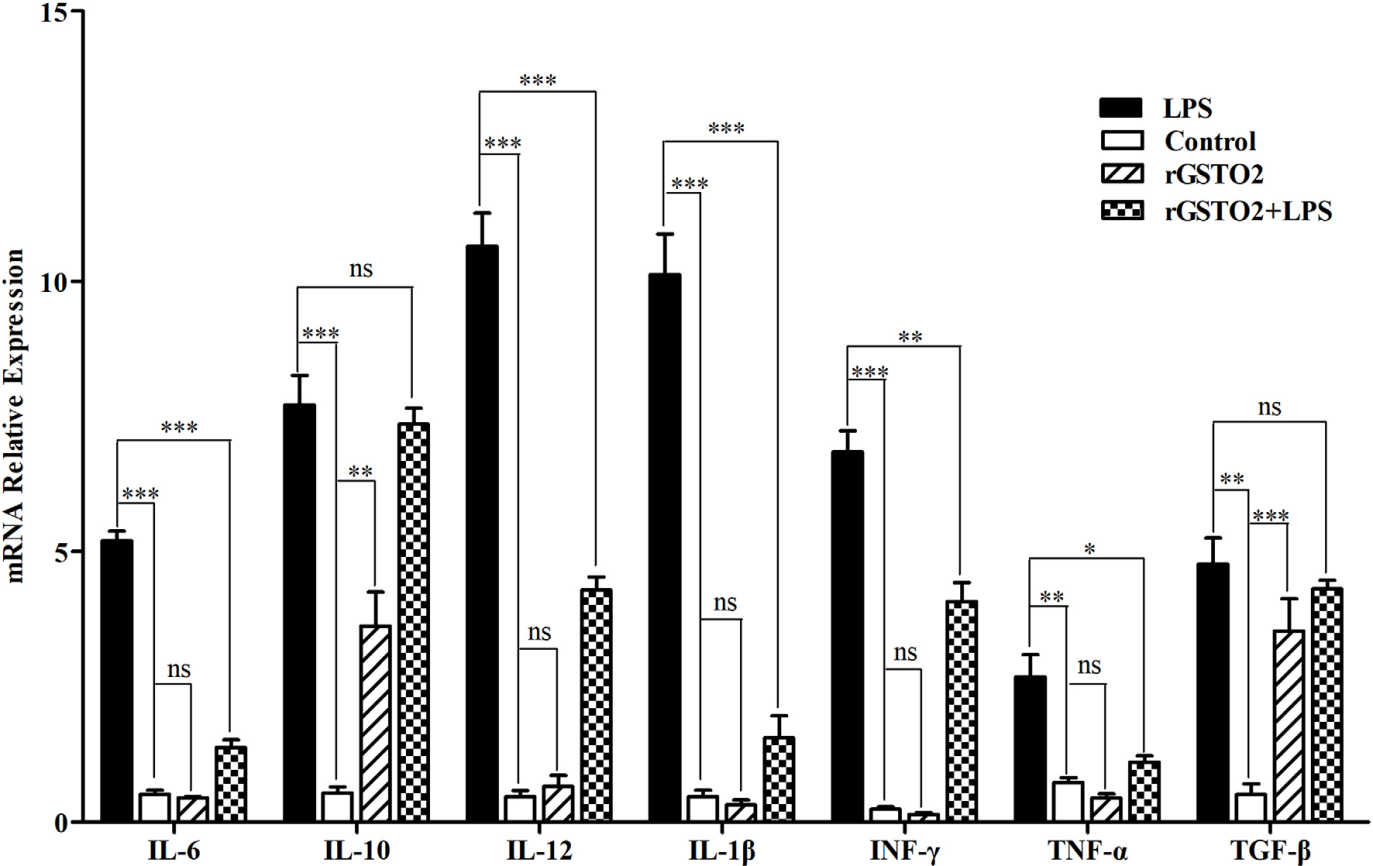
Analysis of the mRNA expression levels of cytokines in RAW264.7 macrophages stimulatd by rGSTO2 protein. Total RNA was extracted from macrophages with rGSTO2 alone or co-incubated with LPS for 24 h, then the mRNA expressions of IL-6, IL-10, Il-12, IL-1β, INF-γ, TNF-α and TGF-β were detected by real-time PCR, respectively. The significance level was set at *p < 0.05, **p < 0.01 and ***p < 0.001 as compared to the control group. All data were expressed as means ± SD (*n* = 3).

The effect of rGSTO2 on the expression of cytokines in LPS-induced RAW264.7 macrophages were examined by real-time PCR. As shown in Figure 7, compared with LPS incubation alone group (positive control), rGSTO2 co-incubation with LPS significantly decreased the mRNA expression levels of macrophage cytokines IL-6 (***p < 0.001), IL-12 (***p < 0.001), IL-1β (***p < 0.001), INF-γ (**p < 0.01) and TNF-α (*p < 0.05). In contrast, the expression of anti-inflammatory cytokine IL-10 (**p < 0.05) and TGF-β (***p < 0.001) mRNA in RAW264.7 macrophages were significantly increased when rGSTO2 was incubated alone as compared with control.

## Discussion

Glutathione transferases (GSTs) are encoded by a large and complex gene superfamily, which are ubiquitous multi-functional enzymes and play important roles in cellular detoxification. On the one hand, GST can bind with GSH and oxidize free radicals to achieve detoxification. On the other, some GSTs can act as glutathione peroxidases to directly exert detoxification. As one of the most important detoxification enzymes in an organism, GSTs play a considerable role in resisting harmful substances, since most helminths lack cytochrome P-450 detoxifying enzymes [33]. Available studies have found that GSTs of worms are involved in the detoxification of lipid peroxides and carbonyl cytotoxins produced by oxygen reactive intermediates, which are derived from endogenous parasite metabolism or host immune system [37]. *Fasciola hepatica* can release multiple immune molecules that including GST protease through different pathways (tegument or ESPs and extracellular vesicles). A recent study has confirmed that sigma-class GST was secreted from *F. hepatica* via extracellular vesicles [11], but the secretion pathway of GSTO protein was still ambiguous. During migration in the peritoneum and penetration through the host liver [10], GSTs are involved in the synthesis of prostaglandin to suppress the host’s protective immune response [9], which is beneficial for long-term parasitism by *F. hepatica* and causes chronic infestation of the host.

Omega GST was purified from cytosolic fractions of adult flukes using GSH- and S-hexyl-glutathione-agarose, separated by 2-DE and identified by MS/MS, and we then made a comparison of the consensus three-element signature of the putative omega class GST in *F. hepatica* with other omega GSTs [9]. It was revealed that the GSTO protein of *F. hepatica* contains an N-terminal glutathione-binding domain and a C-terminal α-helical domain, and there was a highly conserved cysteine residue, which was unique to omega GSTs as compared with other GSTs [19, 40]. Moreover, compared with GSTO1 protein, GSTO2 had some differences in the spatial structure of GSH-binding domain and active site, which implied differences in binding and kinetic constants between GSTO1 and GSTO2 [4, 40]. Furthermore, there are some differences in expression localization of GSTO in different parasites. In *Clonorchis sinensis*, GSTO is mainly expressed in the reproductive system, including yolk follicles, spermatophore and ova [25], whereas GSTO was mainly distributed in the intestinal tract in *Fasciola gigantica* [29]. The present study confirmed that the GSTO2 protein was expressed in yolk follicles, intestinal cells, excretory pores and yolk gland cells of adult *F. hepatica*. Given that GSTO2 was mainly expressed in the reproductive system of the parasite, it was hypothesized that it might help the parasite to resist oxidative stress and protect the reproductive system from stress damage.

Apoptosis has been proven to be an effective mechanism to avoid immune response in helminth infection [15]. Studies have found that ESP released by *F. hepatica* during migration can induce eosinophil and mouse peritoneal macrophage apoptosis *in vitro*, while the apoptosis was related to the induction time and concentration of ESP, which may play a key role in the long-term parasitism of *F. hepatica* in hosts [20]. Here, we analyzed the effect of rGSTO2 protein on the apoptosis of RAW264.7 macrophages *in vitro*, and the results showed that rGSTO2 protein could induce apoptosis of RAW264.7 macrophages in a concentration-dependent manner.

The survival of *F. hepatica* in the host and chronic infestation are inextricably linked to secretion by the parasite of multiple molecules with regulatory effects on host immune response [12, 20, 35]. During infestation, adult *F. hepatica* can release GST (as one of important components of ESPs), which is not only involved in the immune responses of the host, but related to the resistance of *F. hepatica* to triclabendazole, and also reduced liver disease. Therefore, GST is often used as a candidate vaccine or drug target for research [14, 16, 35]. Previous research has reported that the main component of ESPs of this parasite may suppress immune responses of the host by modulating the function of antigen presentation cells (APCs) [30]. As an important APC that triggers host immune defense, macrophages (Mφ) can convert into classically activated macrophages (CAMs) and alternative activated macrophages (AAMs) under different stimulatory conditions [10]. Consequently, Mφ can be activated by pathogen-derived products or host cytokines, and then promote the differentiation of T-helper (Th2) cells, thereby eliciting the production of cytokines IL-4, IL-5 and IL-13 [12]. Recent studies revealed that Mu-class GST from *F. hepatica* could significantly suppress the expression of inflammatory cytokines (IL-1β and TNF-α) in LPS-stimulated murine macrophages *in vitro*, thereby exerting anti-inflammatory activity to prevent the elimination of this worm [2]. To verify whether GSTO2 of *F. hepatica* elicits a similar immune response, we investigated the immunomodulatory effects of rGSTO2 on murine macrophages. It was revealed that LPS-induced mRNA levels of the pro-inflammatory cytokines IL-6, IL-12, IL-1β, INF-γ and TNF-α were significantly inhibited in RAW264.7 macrophages treated by rGSTO2 and LPS together, whereas the expression of the anti-inflammatory cytokines IL-10 and TGF-β were significantly enhanced in rGSTO2-treated macrophages, which may be responsible for the establishment of a regulatory anti-inflammatory Th2 immune response in its host [13]. However, the detailed mechanisms on immunodepression meditated by GSTO2 need to be further deciphered.

In summary, this study for the first time confirmed that *F. hepatica* GSTO2 possesses thioltransferase and dehydroascorbate lyase activity. Meanwhile, rGSTO2 protein could be taken up by murine macrophages and was involved in modulating cell viability and cytokine expression, suggesting that GSTO2 plays an important role in the regulation of physiological functions of host macrophages, which provides new insights into the immune-evasion mechanism of *F. hepatica* and contributes to the development of a potential anti-inflammatory agent.

## Supplementary Material

The Supplementary materials of this article are available at https://www.parasite-journal.org/10.1051/parasite/2022016/olm.**Supplementary Figure 1:**
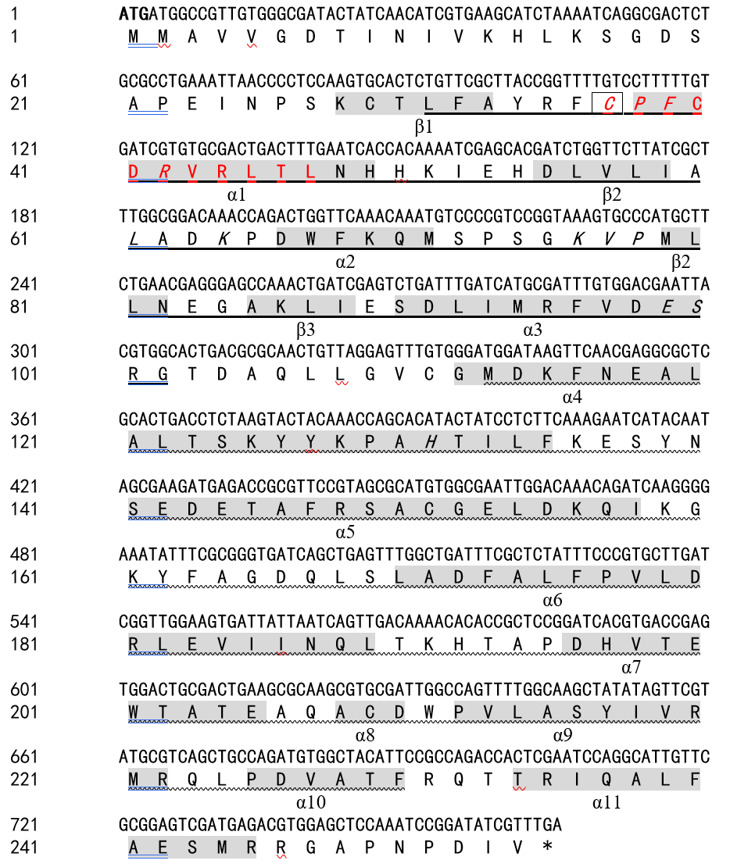
Nucleotide and deduced amino acid sequences of *GSTO2* gene of *F. hepatica.* The start and stop codons are indicated in bold and “*”. The conservative cysteine residues are shown by a box, while the GST_N and GST_C regions are indicated by underline and wave, respectively. The α-helicals, β-strands, glutaredoxin active site and glutathione-binding residues are marked in shadow, red and italic, respectively.
